# Synthesis of some novel 2-(3-cyano -6-(thiophen- 2-yl)-4,4′- bipyridin-2- yloxy)acetohydrazide derivatives: assessment of their cytotoxic activity

**DOI:** 10.1186/s13065-020-00692-4

**Published:** 2020-06-02

**Authors:** Hossa F. Al Shareef

**Affiliations:** Department of Chemistry, Faculty of Applied Sciences, Umm Al-Qura University, P. O. Box 13401, Makkah, 21955 Saudi Arabia

**Keywords:** Pyridine, Schiff bases, Breast cancer, Apoptotic cells, Thiophene

## Abstract

A new series of pyrazole, bipyridine, *N*-amide derivatives and Schiff bases was synthesized using compound 2-(3-cyano-6- (thiophen-2-yl)-4,4′- bipyridin-2-yloxy) acetohydrazide (**3**) as a starting material. The compounds structures were confirmed depending on the spectroscopic methods and elemental analysis. Also, the compounds were evaluated as anticancer agents by the compounds screened towards adenocarcinoma breast cancer cell line (MCF-7). The compounds showed a promising cytotoxic effect against human breast cancer cells. Compound **7c** showed the most effective activity compared to other compounds with (IC_50_ = 0.6 ± 0.01 μg mL^−1^) in comparison with the reference drug doxorubicin (IC_50_ = 1.6 ± 0.02 μg mL^−1^). While compound **3** is closely active with doxorubicin. Also compounds **2**, **4**, **6**, **7a, 7b** and **7d** showed noticeable cytotoxic effect. Early and late apoptotic cells were detected using Acridine orange/Ethidium bromide staining technique. The results of biologically screening of the tested compounds give an idea about the importance in the compounds acting against breast cancer and may lead to the discovery of a potent anticancer agent.

## Introduction

Cancer disease is one of the most widely spread diseases nowadays especially breast cancer. Breast cancer comes in various forms either histological or clinical because it is a heterogeneous disease. Its treatment is done through chemotherapy and/or hormone therapy. Heterocyclic compounds that incorporating pyridine moiety appear miscellaneous pharmacological properties such as anticancer [1], antimicrobial [2, 3], anticonvulsant [4], antiviral [5], anti- HIV [6], antifungal and, antibacterial activities [7]. Also the antitumor activity of pyridine ring enhanced by introducing different substituents such as hydrazide bearing either thiazole, thiophene, benzothiophene, triazole or pyrazole, and cyanoacetohydrazide [8]. Studying Structure-activity relationship (SAR) of the compounds is due to the well-reported anticancer activity of these rings. Compounds containing a pyridine group that includes a cyano group have excellent antitumor activity as reported in the previous publications [9–15]. Based on the reported biological activity of these heterocyclic moieties [16, 17], Schiff bases [18–20], triazoles [21, 22], quinolones and spiro compounds [23, 24] as anticancer agents [25] and continuing of my research on the chemistry of the biologically active compounds [25–30]. Herein, I designed new biologically active compounds using 2-(6′-(4-chlorophenyl) -3′-cyano-3,4′-bipyridin-2′-yloxy) acetohydrazide(**3**) as a building block and studying their antitumor activity against breast cancer cell line.

## Results discussion

### Chemistry

**Scheme 1:**
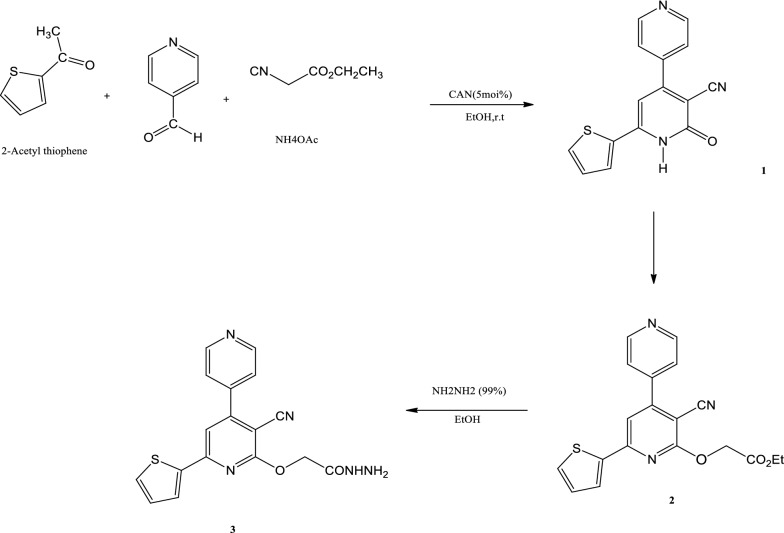
Synthesis of 2-(3-cyano-6- (thiophen-2-yl)- 4,4′-bipyridin- 2-yloxy)acetohydrazide (**3**)

In this research, a one-pot manner was used for the synthesis of compound2-oxo-4-(pyridin-4-yl)-6-(thiophen-2-yl)-1,2-dihydropyridine-3-carbonitrile (1) where all the reaction components, 2-acetylthiophene, 4-pyridine carboxaldehyde, ammonium acetate, and ethyl cyanoacetate were added in the presence of ceric ammonium nitrate (CAN) and then refluxed in ethanol. The resulting compound **1** then alkylated with ethyl bromoacetate in ethanol and in the presence of a catalytic amount of potassium carbonate to give the alkylated derivative ethyl 2-(3-cyano -6-(thiophen-2-yl)-4,4′-bipyridin -2-yloxy)acetate (**2**). The structure of compound **2** was confirmed depending on the spectral data. For example, in the ^1^H NMR spectrum, the characteristic signals of the ethoxy group appeared at 1.18 ppm for (CH_3_) and at 4.15 ppm for (OCH_2_) and the signal for (NH) group at 8.79 ppm was disappeared. Hydrazionlysis of compound **2** gave the acid hydrazide **3**. In the acid hydrazide ^1^H NMR spectrum the signals of the ethoxy groups at 4.15 and 1.18 ppm were disappeared and new signals appeared at 8.75 and 12. 48 for the (NH-NH_2_) group. All other signals appeared at their expected position as illustrated in the experimental section. 2-(3-Cyano-6-(thiophen-2-yl)-4,4′- bipyridin-2-yloxy)acetohydrazide (**3**), is used as a starting matter for the synthesis of all target compounds in this work (Scheme 1).

**Scheme 2:**
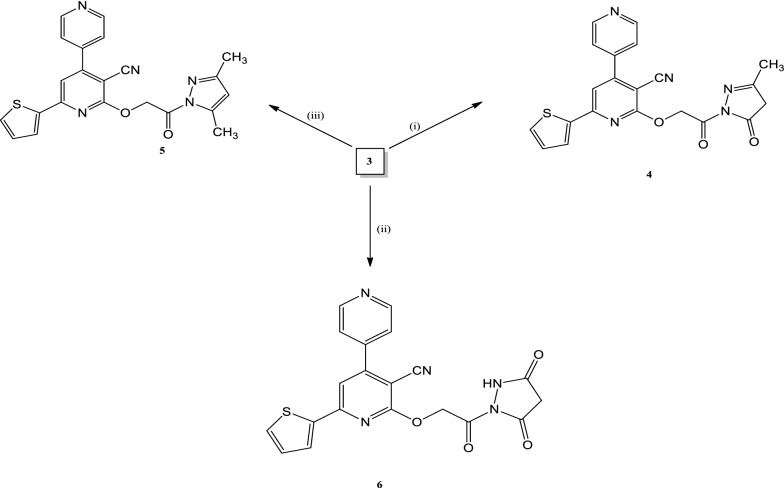
Synthesis of pyrazole derivatives (4-6). Reagents and conditions :(i) ethyl acetoacetate/AcOH,reflux;(ii) diethyl malonate/AcOH or ethyl cyanoacetate/AcOH, reflux; (iii) acetylacetone/AcOH reflux

Compound **3** was cyclized into different heterocyclic moieties. Cyclization of **3** with ethyl acetoacetate and/or acetylacetone gave the corresponding. 2-(2-(3- methyl -5-oxo-4,5-dihydropyrazol-1-yl)-2-oxo ethoxy)-6- (thiophen-2-yl)- 4,4′-bipyridine- 3carbonitrile (**4**) and/or 2-(2-(3,5-dimethyl-1H-pyrazol-1-yl)-2-oxoethoxy)-6- (thiophen-2-yl)-4,4′-bipyridine-3-carbonitrile(**5**), respectively. The compounds’ structures were confirmed based on their spectroscopic data and their elemental analysis wherein both compounds, the characteristic signals of (NH-NH_2_) group disappeared. In compound **4** new signals appeared at 1.84 ppm for (CH_3_) group and at 2.88 for (CH_2_) in pyrazole ring. While in compound **5** new signals at 1.81, 2.01 for (2CH_3_) have appeared. Also in the ^13^C NMR spectra of compound **4** a new signal for the new carbonyl group in pyrazolone ring have appeared. All the appeared signals are in accordance with the expected values. Cyclization of compound **3** with ethyl cyanoacetate or diethyl malonate gave the corresponding 2-(2-(3, 5- dioxopyrazolidin-1-yl)-2-oxoethoxy)-6-(thiophen-2-yl)-4,4′-bipyridine-3-carbonitrile (**6**) (Scheme 2). In the ^1^H NMR spectrum of compound **6** a characteristic signal of (CH_2_) at 2.51 ppm in pyrazolidine ring have appeared.

**Fig. 1:**
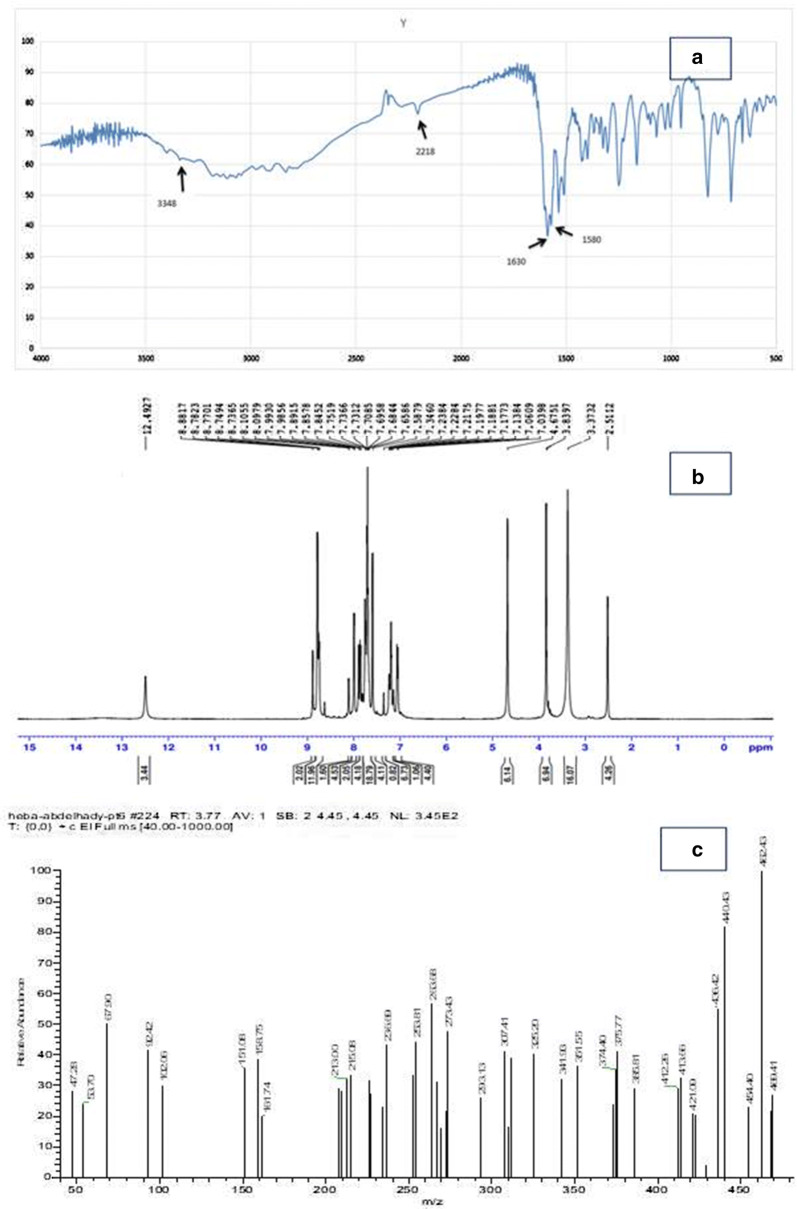
Analysis for the compound **7C**: **a** infrared spectrum, **b** nuclear magnetic resonance spectrum, **c** mass spectrum

**Scheme 3:**
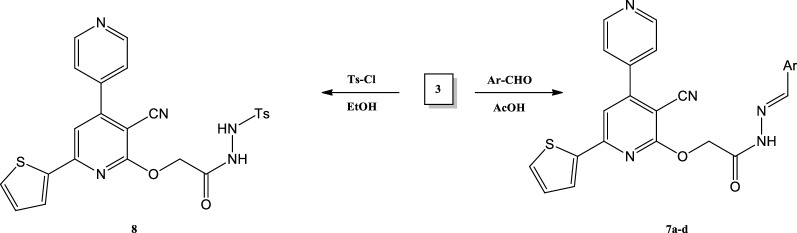
Synthesis of Schiff base **7a**–**d** and compound **8**

A new series of expectedly biologically active *N*-amide derivatives and Schiff bases was synthesized. Schiff bases **7a**–**d** were obtained through condensation of compound **3** with different aldehydes namely 3-pyridine carboxaldehyde, 3,4- Diydroxy benzaldehyde, anisaldehyde and vanillin, in acetic acid. In all Schiff bases the signal characteristic to the (NH_2_) group was disappeared and the signal of (NH) group at 12.48 ppm was shifted to new positions at 12.48, 9.99, 12.49 and 8.84 ppm. In compounds **7a**, **7b**, **7c**, and **7d** respectively. All the characteristic signals of the arylidine groups were appeared at their expected positions as shown in the experimental part, Compound **7c** structure was confirmed based on the spectroscopic data in (Fig. 1) The reaction of **3** with p-toluenesulfonyl chloride in absolute ethanol afforded the corresponding 2-(3-cyano-6(thiophen-2-yl)-4,4″-bipyridin-2-yloxy)*N*-(tosylmethylene)aceto hydrazide (8) (Scheme 3). Compound **8** structure was confirmed based on the spectroscopic data and the elemental analysis.

### In vitro anticancer screening

**Fig. 2:**
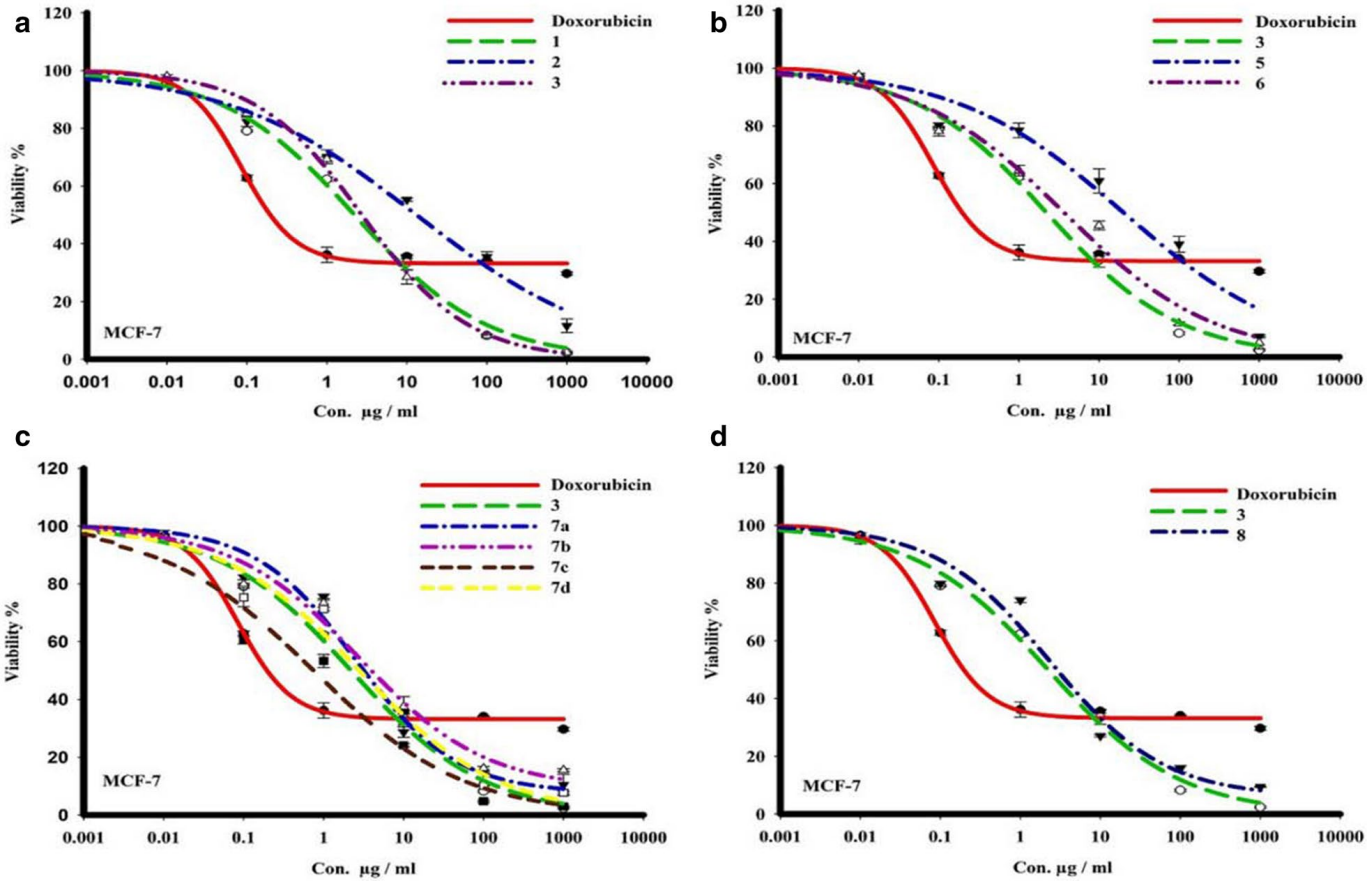
The dose response curves of the cytotoxicity of different compounds towards MCF-7tumor cell line. Cells were exposed to plant extract with different concentrations for 72 h. Cell viability was determined by SRB stain

The in vitro cytotoxic activities of compounds **1**, **2**, **3**, **5**, **6**, **7a**–**d** and 8 were determined using SRB assay towards breast cancer cell line (MCF-7) over concentration range of 0.01 to 1000 μg. The tested compounds exhibited a variable cytotoxicity profile against the tested human breast cancer cells. (Table 1 and Fig. 2). doxorubicin is a reference drug in this study The IC_50_: is the compounds concentrations reduce the cell viability to 50%. The data in Table 1 and Fig. 2 indicate the cytotoxicity profile of the newly synthesized compounds against breast cancer cells. The results showed considerable cytotoxicity against cancer cell, most of the compounds showed highly cell killing significant on MCF-7 cells; some of them were revealed a strong activity, others were found to be on par near the reference drug toxicity (IC_50_ =  1.3 μg mL^−1^).

**Table 1:** The IC_50_ (µg mL^−1^) of the compounds 1, 2, 3, 5, 6, 7a–d, 8 against breast cancer (MCF-7) cell line

Compounds	MCF-7 IC_50_ (µg)
**Dox.**	1.6 ± 0.02
**1**	4 ± 0.18
**2**	8.2 ± 0.7
**3**	1.3 ± 0.04
**5**	28.7 ± 1.4
**6**	4.7 ± 0.3
**7a**	3.5 ± 0.2
**7b**	4.4 ± 0.2
**7c**	0.6 ± 0.01
**7d**	2.7 ± 0.04
**8**	3 ± 0.2

The Schiff base **7c** (IC_50_ =  0.6 μg mL^−1^) is the most potent compound in this evaluation and it showed higher activity than doxorubicin itself; then the sulphonamide derivative **3** with (IC_50_   =  1.3 μg mL^−1^) have activity nearly to the reference drug. Compounds **1**, **3**, **6**, **7a**, **7b**, and **7d** had a highly toxic effect against breast cancer cell with IC_50_s ranging from 1.3 to 4.7 μg mL^−1^ compared to doxorubicin, and the compound **2** has a moderate cytotoxic effect with IC_50_ = 8.2 μg mL^−1^. While compound **5** has a weak activity with IC_50_ = 28.7 μg mL^−1^ compared with other compounds and compared to doxorubicin.

After staining cells using double stains AO/EtBr, cells appeared in the form of four colors as follows: living cells (normal green nuclei), early-programmed cell death (apoptotic) (bright green nucleus with segmented chromatin), late-programmed cell death (apoptotic) (orange nucleus with chromatin condensation or fragmentation) and necrotic cells (Kernel of uniformly colored orange cells).

**Fig. 3:**
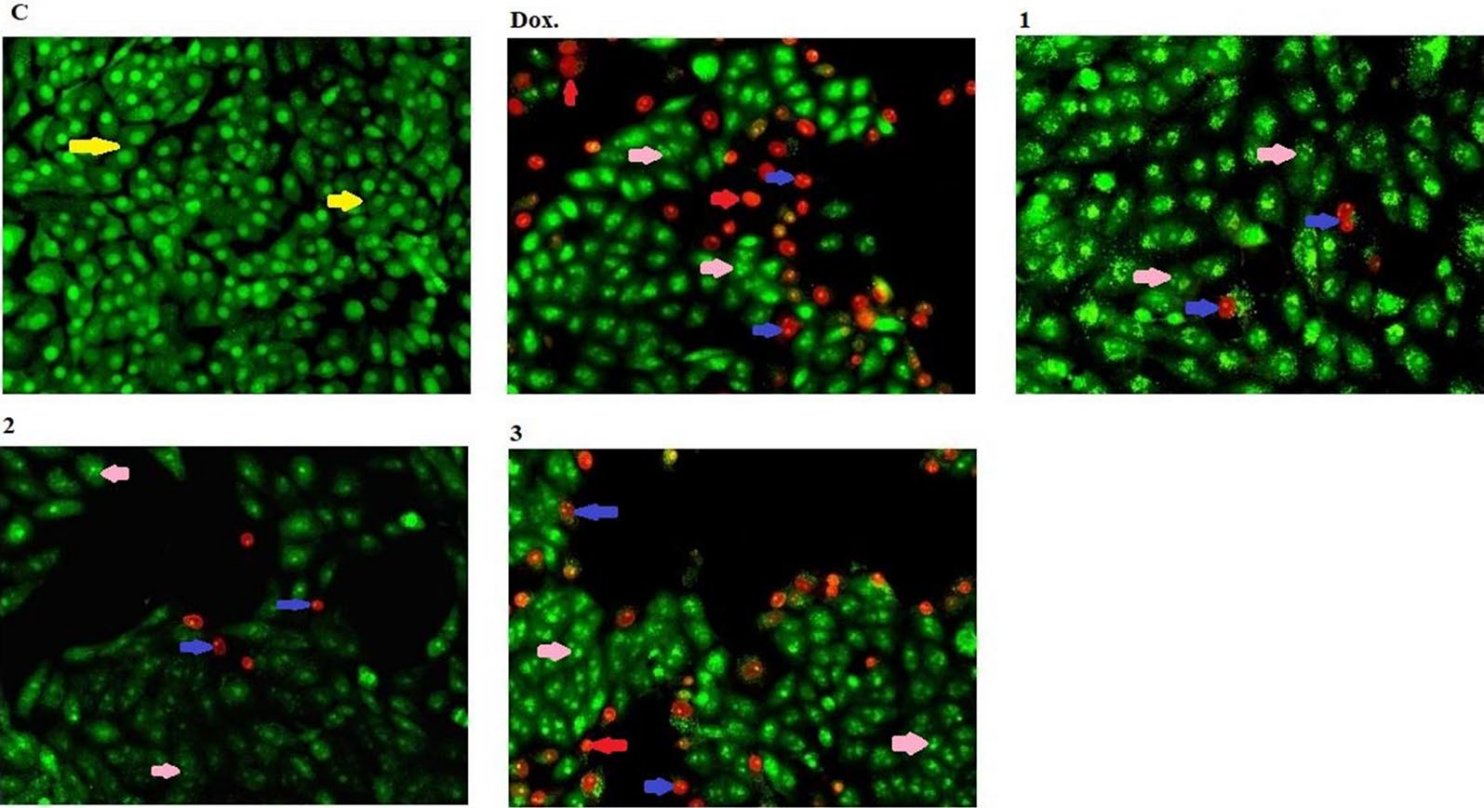
Nuclear morphological conersions of MCF-7 cells, after treated using chemical compounds 1, 2, and 3 compared with reference drug doxorubicin. Compounds stimulate different nuclear changes such as condensation and fragmentation of chromatin, nuclei condensation, as demonstrated by acridine orange/Ethidium bromide staining at 200×. Yellow arrows indicate live cell, pink arrows indicate early apoptotic Red arrows indicate necrotic and blue arrows indicate late apoptotic cells

**Fig. 4:**
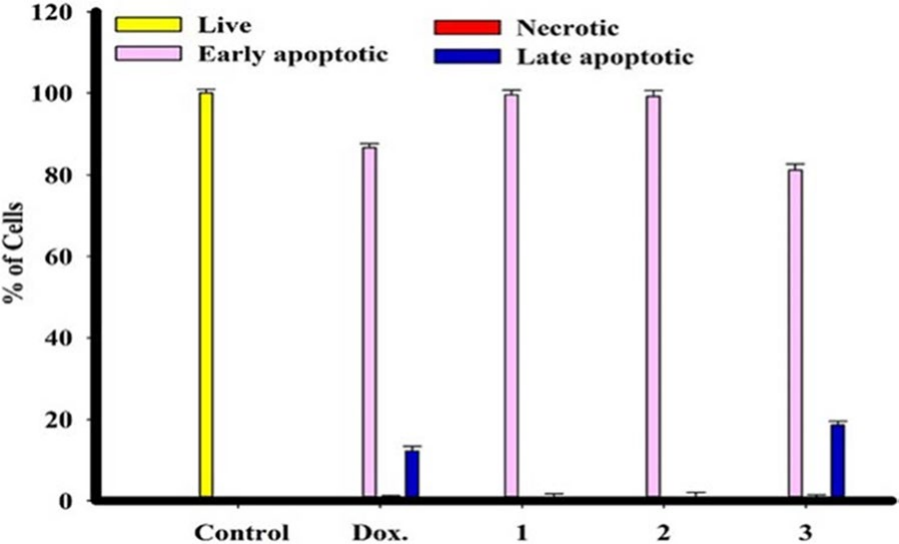
Rate of apoptotic MCF-7 tumor cells after 48 h treatment with chemical compounds (mean ± SD of three independent experiments in three repeats each) compared to cell control

The uniformly stained green cells with normal, round and intact nuclei that indicates the healthy cell control. Whereas, the highly cell killing with late apoptotic observed by treatment with compound **1** and some necrotic cell also observed with the compound itself; on the other hand there are no necrotic cells with compounds **2** and **3** compared to compound **1**, and the derivative acetohydrazide **3** have high rate of late apoptotic compared to compounds **1** and **2** (Figs. 3 and 4).

**Fig. 5:**
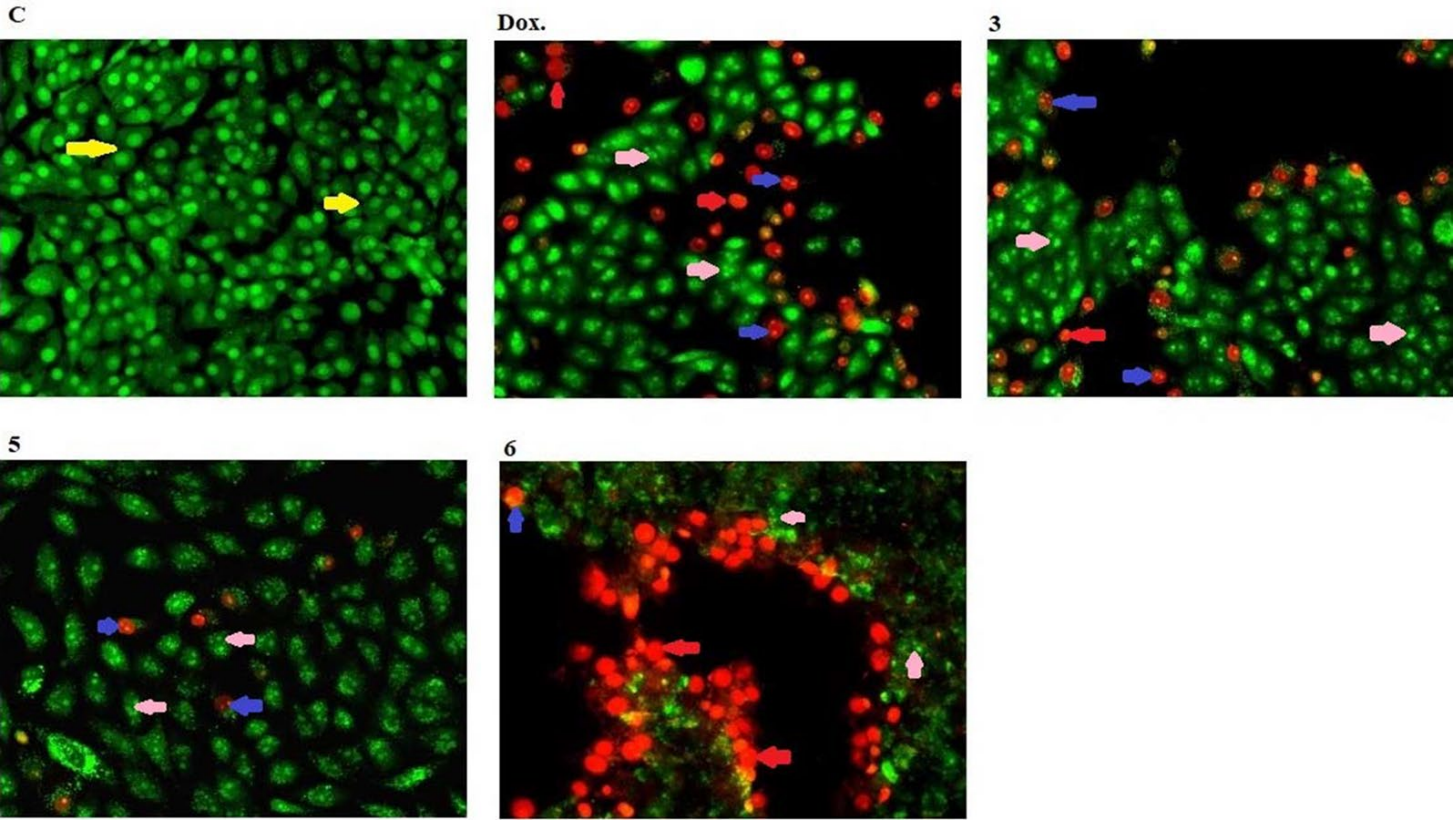
Morphological and nuclear changes of MCF-7, tumor cells after treatment by chemical compounds 3, 5, and 6 compared with reference drug doxorubcin. Compounds induced various nuclear features such as chromatin fragmented and condensation, nuclei condensation, as demonstrated by acridine orange/Ethidium bromide staining at 200 × . Yellow arrows indicate live cell, pink arrows indicate early apoptotic Red arrows indicate necrotic and blue arrows indicate late apoptotic cells

**Fig. 6:**
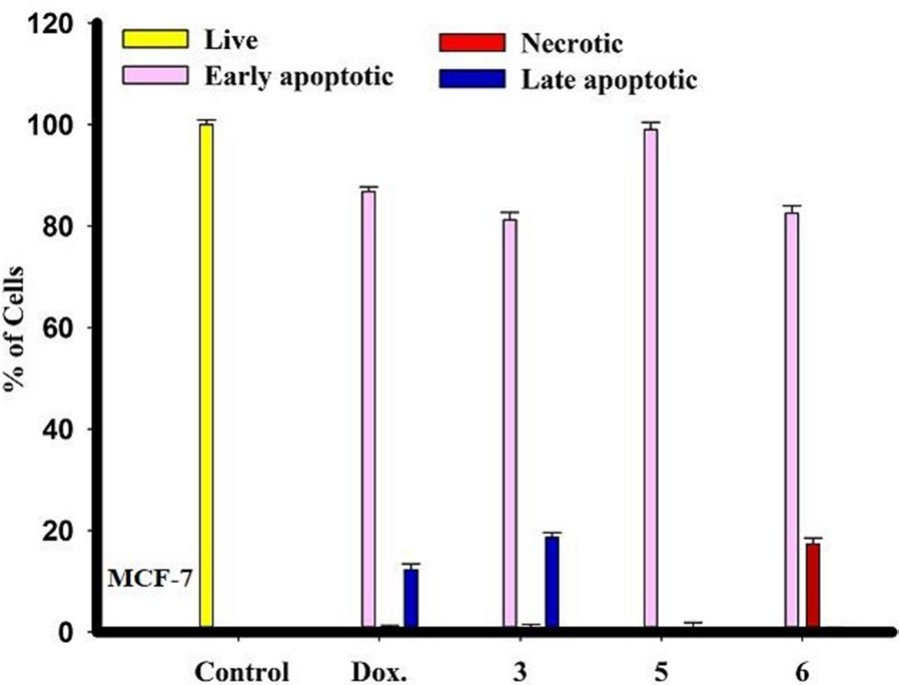
Rate of apoptotic MCF-7 tumor cells after 48 h treatment with chemical compounds (mean ± SD of three independent experiments in three repeats each) compared to cell control

The compound **5** killing the cells with early apoptotic way was more pronounced compared to compounds **3** and **6**. Compound **6** has a necrotic cells after treatment compared to compounds **3** and **5**. Also, compound **3** have cells with late apoptotic more than compounds **5** and **6** (Figs. 5 and 6).

**Fig. 7:**
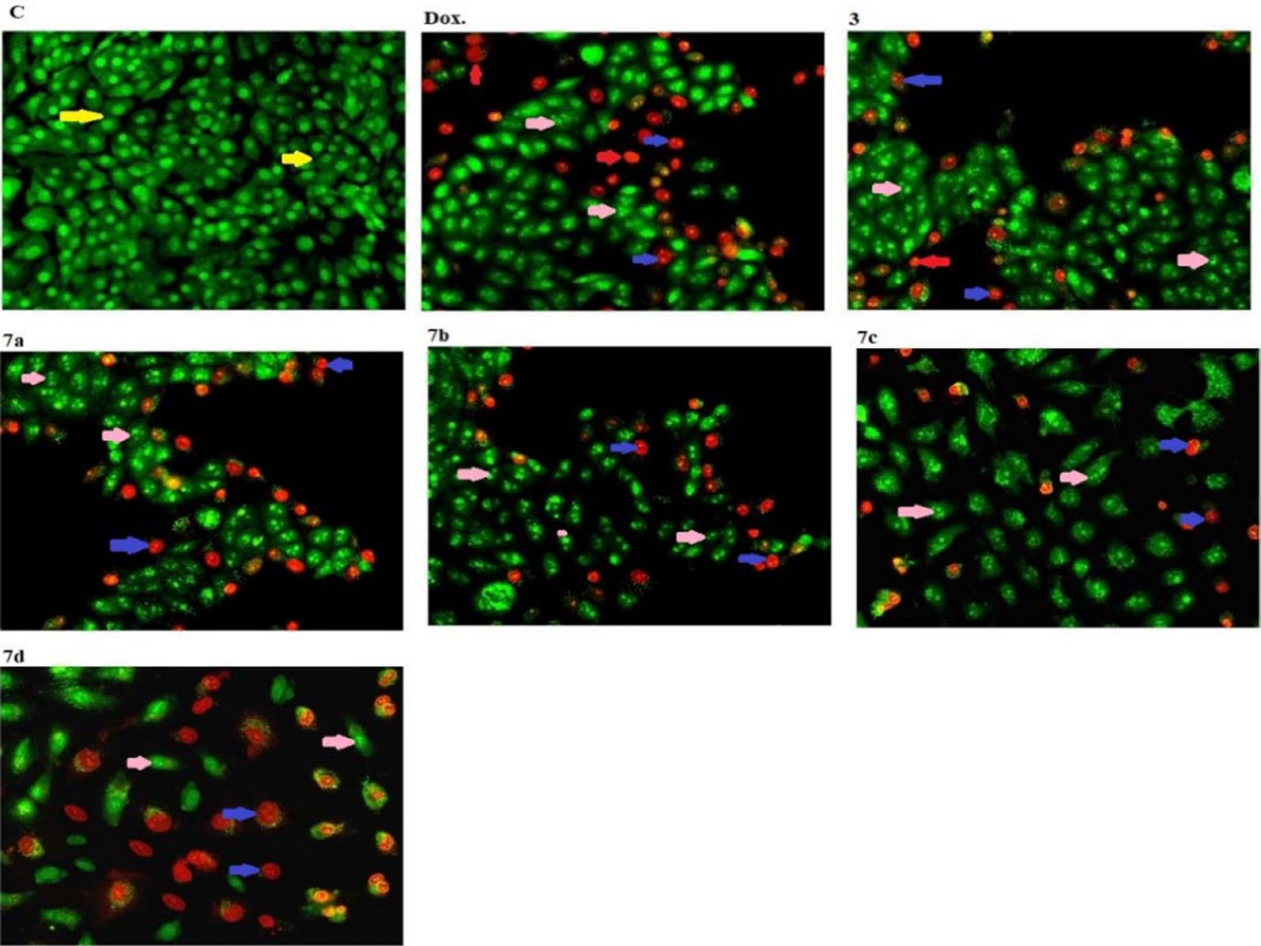
Morphological and nuclear changes of MCF-7, tumor cells after treatment by chemical compounds 3, 7a, 7b, 7c and 7d compared with reference drug doxorubcin. Compounds induced various nuclear changes such as chromatin fragmented and condensation, nuclei condensation, as demonstrated by acridine orange/Ethidium bromide staining at 200×. Yellow arrows indicate live cell, pink arrows indicate early apoptotic Red arrows indicate necrotic and blue arrows indicate late apoptotic cells

**Fig. 8:**
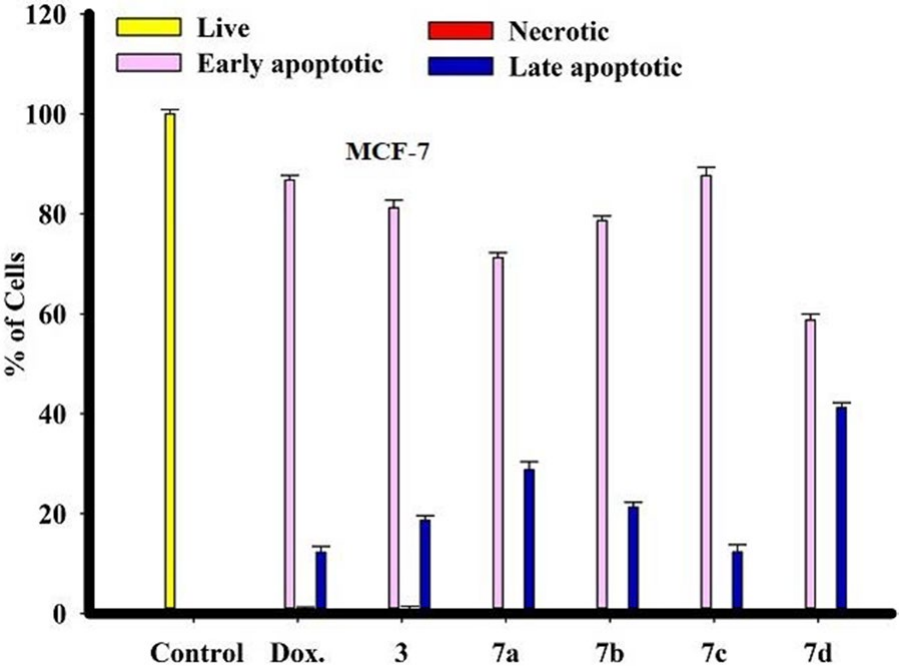
Rate of apoptotic MCF-7 tumor cells after 48 h treatment with chemical compounds (mean ± SD of three independent experiments in three repeats each) compared to cell control

Compound **7d** has a highly late apoptotic effect on cancer cells compared to **3**, **7a**, **7b**, **7d** and compound **7c** then **3** have early apoptotic more than **7a**, **7b**, and **7d** is lower (Figs. 7 and 8).

**Fig. 9:**
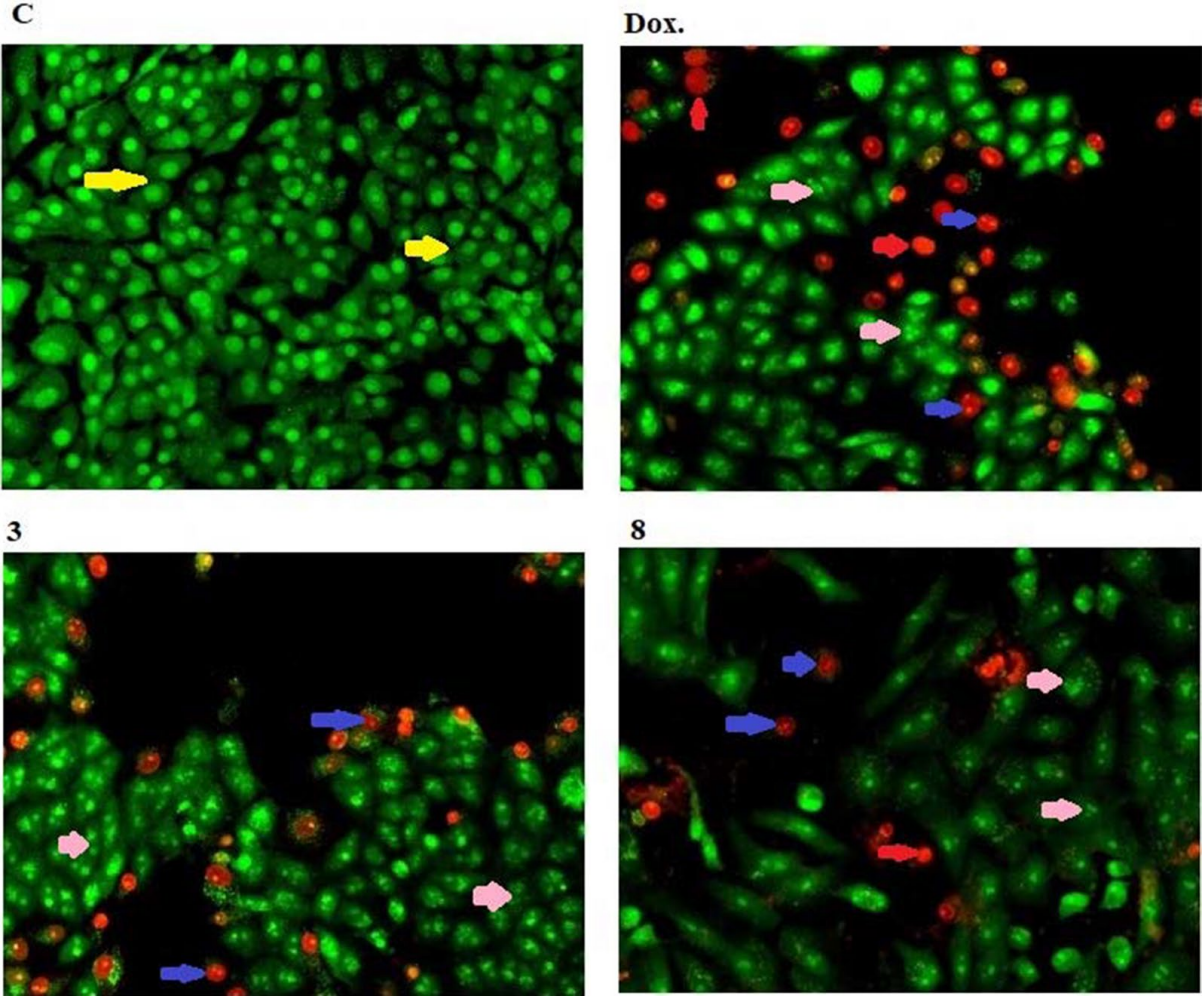
Morphological and nuclear changes of MCF-7, tumor cells after treatment by chemical compounds 3, and 8 compared with reference drug doxorubcin. Compounds stimulate different nuclear changes such as chromatin fragmented and condensation, nuclei condensation, as demonstrated by acridine orange/Ethidium bromide staining at 200×. Yellow arrows indicate live cell, pink arrows indicate early apoptotic Red arrows indicate necrotic and blue arrows indicate late apoptotic cells

**Fig. 10:**
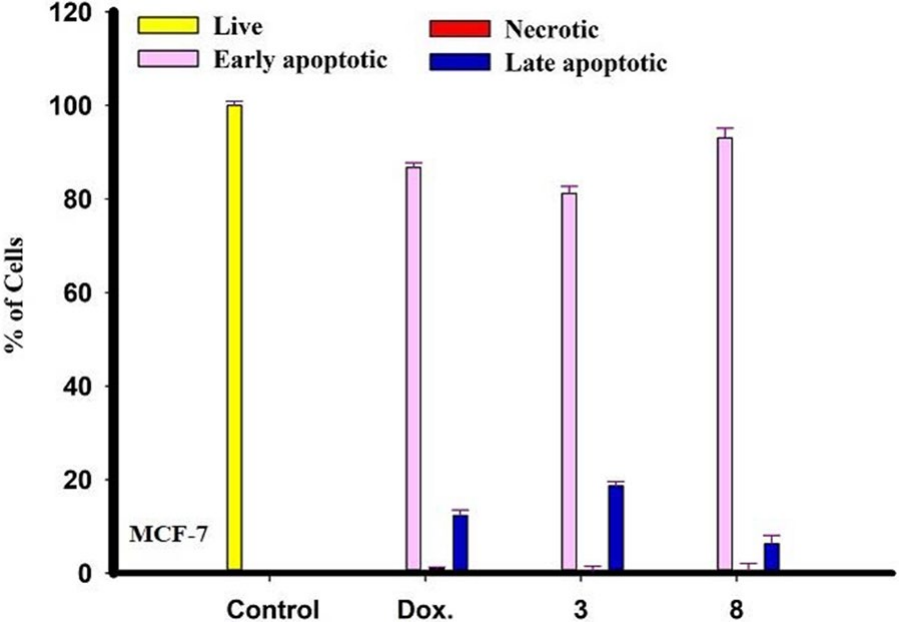
Rate of apoptotic MCF-7 tumor cells after 48 h treatment with chemical compounds (mean ± SD of three independent experiments in three repeats each) compared to cell control

Whereas, compound **8** has early apoptotic killing effect and cell necrotic against cancer cells more than compound **3**, while compound **3** has a more cell late apoptotic effect than compound **8** (Figs. 9 and 10).

The biological activity of the tested compounds were indicated the promising cell killing effect of the 4,4′ bipyridine moiety in the compounds acting towards breast tumor cells.

## Conclusions

In this paper I used compound 2-(3-cyano-6-(thiophen-2-yl)-4,4′-bipyridin -2-yloxy)acetohydrazide **3** to synthesis a novel substituted pyrazole, bipyridine, *N*-amide derivatives and Schiff bases. The anticancer activity of the compounds was assessed against breast cancer cell line (MCF-7). The data obtained for the tested compounds shows the possible importance of these compounds to act as anticancer agents where compound **7c** showed better activity than the standard drug itself. While other compounds such as compound **3** is equipotent with the standard drug. Compounds **2**, **4**, **6**, **7a**, **7b** and **7d** showed obvious activities but less than the reference.

## Materials and methods

### Chemistry

Melting points were measured on a Gallenkamp apparatus, and are uncorrected. The desired time for completing the reaction was monitored by TLC. The IR spectra were recorded using (KBR) plates on a Shimadzu 470 IR spectrometer. The ^1^H and ^13^C NMR spectra were measured on a Bruker 400DRX-Avance NMR spectrometer at 400 MHz and chemical shifts (δ) are in ppm relative to TMS (tetramethylsilane). Mass spectra were measured on GC/MS with electron impact ionization by to (70 eV). Elemental analyses were performed on Perkin-Elmer 2400 series П CHN elemental analyser.

#### Synthesis of 2-oxo-4-(pyridin-4-yl) -6-(thiophen-2-yl) -1,2- dihydro pyridine-3-carbonitrile (1)

4-Pyridine carboxaldehyde (0.01 mol), 2-acetyl thiophene (0.01 mol), ethyl cyanoacetate (0.01 mol), ammonium acetate (0.15 mol) and 5 mol% of CAN in ethanol (25 mL) in a 50 mL round-bottom flask were refluxed for 2 h. After completion of the reaction, the solid product obtained was collected, filtered, washed several times by water dried and then crystallized from ethanol to give compound **1** as yellow crystals in yield 89%, m.p. 205 °C. IR (KBr): 3093 (NH), 2218 (CN), 1673 (C = O) cm^−1^. ^1^H NMR (DMSO-d6) δ: 7.24–8.67 (m, 8H, Ar–H, thiophene and pyridine rings), 8.79 (s, 1H, NH) ppm. ^13^C NMR (DMSO-d6) δ: 163.53 (C =O), 162.81, 150.71 (2C), 143.52 (2CH), 142.89, 142.32 (2C), 131.68, 129.59, 129.01 (3CH), 122.50 (2CH), 121.9 (C), 116.14 (CN), 113.38 (CH) ppm. MS: m/z (%): 279 (M + , 20), 224 (100). Anal. Calc. (%) for C_15_H_9_N_3_OS: C, 64.50; H, 3.25; N, 15.04; S, 11.48. Found: C, 64.55; H, 3.18; N, 15.11; S, 11.45.

#### Synthesis of ethyl 2-(3-cyano-6-(thiophen-2-yl)-4,4′-bipyridin-2-yloxy) acetate (2)

A mixture of compound **1** (0.01 mol), ethyl bromoacetate (0.01 mol), and anhydrous potassium carbonate (0.15 mol) in acetone was refluxed for 2 h. After completion of the reaction the mixture was poured onto the ice, the product separated was collected by filtration, dried, and crystallized from ethanol to give **2** as pale yellow needles in yield 75%, m.p. 159–160 °C. IR (KBr): 2224 (CN), 1753 (C =O), 1600 (C=N) cm^−1^. ^1^H NMR (DMSO-d6) δ: 1.18 (t, 3H, J = 6.8, CH_3_), 4.15 (q, 2H, J=6.8, OCH_2_), 5.06 (s, 2H, CH_2_), 7.24-8.82 (m, 8H, Ar–H, thiophene and pyridine rings) ppm. ^13^C NMR (DMSO-d6) δ: 163.26(C = O), 153.48 (C), 150.71 (C), 143.38 (2CH), 142.38 (C), 132.59, 129.88, 129.64 (3CH), 123.51 (2CH), 114.95 (CN), 113.14 (CH), 91.57 (2C), 64.25 (OCH_2_), 61.29 (CH_2_), 14.65 (CH_3_) ppm. MS: m/z (%): 365 (M + , 100). Anal. Calc. (%) for C_19_H_15_N_3_O_3_S. C, 62.45; H, 4.14; N, 11.50; S, 8.77. Found: C, 62.48; H, 4.19; N, 11.45; S, 8.68.

#### Synthesis of 2-(3-cyano-6-(thiophen-2-yl) -4,4′-bipyridin-2-yloxy) aceto hydrazide (3)

A mixture of hydrazine hydrate (99%, 0.04 mol), and compound **2** (0.01 mol), was refluxed in 20 mL absolute ethanol for 5 h. The reaction mixture was poured on an ice-water. The product formed was filtered of, washed with water, dried, and crystallized from ethanol to give **3** as yellow crystals in yield 65%, m.p 226 °C. IR (KBr): 3402.43, 3334.92 (NH_2_), 3267 (NH), 2212 (CN), 1741 (C =O), 1620 (C=N) cm^−1^. ^1^H NMR (DMSO-d6) δ: 4.67 (s, 2H, CH_2_), 7.18–8.58 (m, 8H, Ar–H, thiophene and pyridine rings), 8.75 (d, 2H, NH_2_), 12.48(s, 1H, NH) ppm. ^13^C NMR (DMSO-d6) δ: 160.82 (C =O), 153.20, 153.16 (2C),150.53 (2CH), 143.38 (2C), 143.10(C), 131.25, 129.45, 129.19 (3CH) 123.47 (2CH), 116.33 (CN), 111.44 (CH), 101.86 (CH), 85.76 (C), 56.50 (OCH_2_) ppm. MS (m/z, %): 351 (M + , 20), 101 (100). Anal. Calc. (%) for C_17_H_13_N_5_O_2_S. C, 58.11; H, 3.73; N, 19.93; S, 9.12. Found: C, 58.17; H, 3.68; N, 19.96; S, 9.18.

#### General procedure for the synthesis of compounds 4-6

An equimolar amount of ethyl acetoacetate, acetylacetone and/or ethyl cyanoacetate (or diethyl malonate) and a mixture of compound **3** (0.01 mol) was refluxed in 15 mL acetic acid for 5 h. The produced product after cooling was filtered off, washed with water, dried, and crystallized with acetic acid to give compounds **4, 5,** and **6** respectively.

##### 2-(2-(3-methyl-5-oxo-4,5-dihydropyrazol-1-yl)-2-oxoethoxy)-6-(thiophen-2-yl)-4,4′-bipyridine-3 carbonitrile (4)

Pale yellow crystals in yield 71%, m.p. 202–204 ℃. IR (KBr):2347.37 (CN), 1670.35 (C =O),1637.56(C =O),1620 (C=N)cm^−1^. ^1^H NMR (DMSO-d6) δ: 1.84 (s, 3H, CH_3_), 2.88 (s, 2H, CH_2_), 4.67(s, 2H, CH_2_), 8.78–7.19 (m, 8H, Ar–H, thiophene and pyridine rings) ppm. ^13^C NMR (DMSO-d6) δ: 169.17 (C =O), 167.57 (C=O), 150.38 (C), 147.72 (C), 124.10 (2CH), 144.94 (2CH), 144.78 (C), 129.45, 128.97, 127.44 (3CH), 124.10 (CH), 111.44 (CN), 101.86 (CH), 58.16 (OCH_2_), 42.59 (CH_2_), 22.88 (CH_3_) ppm. MS: m/z (%): 417.03 [M + , 17], 293 (100).Anal.Calc.(%) for C_21_H_15_N_5_O_3_S.C, 60.42; H, 3.62; N, 16.78; S, 7.68. Found C, 60.47; H, 3.68; N, 16.83; S, 7.65,

##### 2-(2-(3,5-dimethyl-1H-pyrazol-1-yl)-2-oxoethoxy)-6-(thiophen-2-yl)-4,4′-bipyridine-3-carbonitrile (5)

Pale yellow crystals in yield 50%, m.p. 197–198 °C.IR (KBr): 3265 (NH), 2213 (CN), 1745 (C =O), 1619 (C=N) cm^−1^. ^1^H NMR (DMSO-d6) δ: 1.81 (s, 3H, CH_3_), 2.01 (s, 3H, CH_3_), 4.66 (s, 2H, CH_2_), 6.21-8.75 (m, 9H, CH pyrazole, pyridine and thiophene rings) ppm. ^13^C NMR (DMSO-d6) δ: 162.02 (C =O), 147.78 (C), 145.72 (C), 144.94 (2CH), 144.50 (C) 143.39(2CH), 128.54, 128.06, 127.45 (3CH), 123.85 (CH), 111.44 (CN), 101.86 (CH), 57.37 (OCH_2_), 15.31 (CH_3_) ppm. MS: m/z (%): 415 [M + , 7], 293 (100). Anal.Calc. (%) for C_22_H_17_N_5_O_2_S.;C,63.60;H,4.12;N,16.86;S,7.72. Found C 63.65; H 4.18; N 16.83; S, 7.77.

##### 2-(2-(3,5-dioxopyrazolidin-1-yl)-2-oxoethoxy)-6-(thiophen-2-yl)-4,4′-bipyridine-3-carbonitrile (6)

Pale yellow crystals in yield 69%, m.p 216–217 °C. IR (KBr): 3400 (NH), 2223 (CN), 1718 (C = O), 1701(C = O), 1617(C=N) cm^−1^.^1^H NMR (DMSO-d6) δ: 2.51 (s, 2H, CH_2_) pyrazoldine) 4.66 (s, 2H, CH_2_), 7.21-8.77 (m, 8H, Ar–H, thiophene and pyridine rings), 10.10 (s, 1H, NH) ppm. ^13^C NMR (DMSO-d6) δ: 175.18 (C=O), 170.05 (2C=O), 153.18 (C =N), 150.38 (C), 144.60 (2CH), 143 (C), 138.34 (C), 129.09, 128.98, 127.44 (3CH), 113.50 (CN), 111.41 (CH), 101.81 (CH), 52.56 (OCH_2_), 22.65 (CH_2_) ppm. MS: m/z (%): 419 [M + , 20], 292 (100). Anal.Calc.(%) for C_20_H_13_N_5_O_4_S.C, 57.28; H, 3.12; N, 16.70; S, 7.664. Found C, 57.33; H, 3.16; N, 16.75; S, 7.69.

#### General procedure for synthesis of Schiff bases 7a-d

A mixture of compound **3** (0.01 mol) and the appropriate aromatic aldehyde (3-pyridincarboxaldehyde, 3, 4 -dihydroxy benzaldehyde, anisaldehyde and vanillin (0.01 mol) in the presence of a catalytic amount of pipredine, in absolute ethanol (10 mL) was refluxed for 2 h. After cooling, the formed precipitate was filtered off, dried, and crystallized from acetic acid to afford the corresponding Schiff base **7a-d**.

##### (E)-2-(3-cyano-6-(thiophen-2-yl)-4,4′-bipyridin-2-yloxy)-N’-(pyridin-3-ylmethylene)acetohydrazide (7a)

Pale Yellow crystals in yield 75%, m. p 189–190 °C. IR (KBr): 3337 (NH), 2347.37 (CN), 1655 (C =O), 1595 (C =N) cm^−1^. ^1^H NMR (DMSO-d6) δ: 4.66 (s, 2H, CH_2_), 8.7–7.18 (m, 13H, Ar–H, thiophene, pyridine and CH alphatic), 12.48 (s, 1H, NH) ppm. ^13^C NMR (DMSO): δ = 162.03 (C=O), 153.16 (C = N), 151.56 (C) 150.38 (C), 149.67 (C), 147.73 (CH), 144.95 (CH), 144.77 (C), 143.10 (CH), 129.46, 128.97, 127.45 (3CH), 124.10 (3CH), 111.44 (CN), 101.87 (CH), 54.99 (OCH_2_) ppm.MS: m/z (%): 440[M + , 20], 374 (100).Anal.Calc. (%)for C_23_H_16_N_6_O_2_S. C, 62.72; H, 3.66; N, 19.08; S, 7.28. Found C,62.76; H,3.70; N,19.11; S, 7.32.

##### (E)-2-(3-cyano-6-(thiophen-2-yl)-4,4′-bipyridin-2-yloxy)-N’-(3,4-dihydroxybenzylidene)acetohydrazide (7b)

Brown crystals in yield 71%, m.p.198–199 °C.IR (KBr):  3400 (OH), 3179(NH), 2347 (CN), 1655, (C =O), 1612 (C =N) cm^−1^. ^1^H NMR (DMSO-d6) δ: 4.67 (s, 2H, CH_2_), 7.19–8.78 (m, 12H, Ar–H, thiophene, pyridine and CH aliphatic), 9.99 (s, 1H, NH), 12.49 (br. s, 1H, OH), 13.71 (br. s, 1H, OH) ppm. ^13^C NMR (DMSO): δ = 169.99 (C=O), 153.17 (C=N), 152.91 (C), 152.03 (C), 151.56 (C), 149.79(2CH), 144.50 (CH), 143.10 (C), 143.10 (CH), 129.11, 128.97, 128.10 (3CH), 124.10 (3CH), 111.44 (CN), 101.86 (CH), 52.57 (OCH_2_) ppm. MS: m/z (%): 471 [M + ,19], 293 (100). Anal.Calc. (%) for C_24_H_17_N_5_O_4_S.C, 61.14; H, 3.63; N, 14.85; S, 6.80.Found C, 61.18;H, 3.67; N, 14.89; S, 6.84.

##### (E)-2-(3-cyano-6-(thiophen-2-yl)-4,4′-bipyridin-2-yloxy)-N’-(4-methox ybenzyli dene) cetohydrazide (7c)

Pale Yellow crystals in yield 85%, m.p180–181 °C. IR (KBr): 3348 (NH), 2218 (CN), 1630 (C = O), 1580 (C = N) cm^−1^. ^1^H NMR (DMSO-d6) δ: 3.83(s, 3H, CH_3_), 4.67 (s, 2CH, CH_2_), 8.88–7.03 (m, 13H, Ar–H, thiophene, pyridine and CH aliphatic), 12.49 (s, 1H, NH) ppm. ^13^C NMR (DMSO): δ = 162.74 (C=O), 153.63 (C=N), 153.17 (C), 152.23 (C), 151.56 (C), 149.58 (2CH), 144.22 (CH), 143.27 (C), 143.10 (CH), 128.97, 128.06, 127.44 (3CH), 123.37 (2CH), 111.44 (CN), 107.84 (CH), 101.87 (CH), 55.91 (OCH_2_), 47.13 (OCH_3_) ppm. MS: m/z (%): 469 [M + , 27], 462 (100). Anal. Calc. (%) for C_25_H_19_N_5_O_3_S. C, 63.95; H, 4.08; N, 14.92; S, 6.83.Found C, 63.91; H, 4.11; N, 14.96; S, 6.87.

##### (E)-2-(3-cyano-6-(thiophen-2-yl)-4,4′-bipyridin-2-yloxy)-N’-(4-hydroxy-3 methoxy benzylidene) acetohydrazide (7d)

Pale Yellow crystals in yield 80%, m.p.240–241 °C. IR (KBr): 3402.43 (OH), 3339 (NH), 2222 (CN), 1654.92 (C =O), 1618 (C =N) cm^−1^. ^1^H NMR (DMSO - d6) δ: 1.49 (s, 3H, CH_3_), 4.67 (s, 2H, CH_2_), 8.78 -6.88 (m, 12H, Ar–H, thiophene, pyridine and CH aliphatic), 8.84 (s, 1H, NH), 12.58 (br. s, 1H, OH) ppm. ^13^C NMR (DMSO):δ = 160.33 (C=O), 153.60 (C=N), 153.17 (C), 152.16 (C), 151.82 (C), 151.56 (C), 149.56 (2CH), 148.68 (C), 147.72 (CH), 144.78 (C), 144.42 (CH), 129.10, 128.97, 127.99 (3CH), 123.38 (2CH), 115.80 (CH), 113.53 (CH), 111.44 (CN), 106.31 (CH), 101.86 (C), 55.81 (OCH_2_), 44.04 (OCH_3_) ppm. MS: m/z (%): 485 [M + , 14], 306.76 (100).Anal.Calc. (%)forC_25_H_19_N_5_O_4_S.C, 61.85; H, 3.94; N, 14.42; S 6.60 found C 61.88; H 3.89;N 14.45; S 6.56.

#### Synthesis of (E)-2-(3-cyano-6(thiophen -2-yl)-4,4″- bipyridin-2-yloxy) N-(tosylmethylene)acetohydrazide (8)

A mixture of p-toluenesulfonyl chloride (1 mmol) and compound 3 (1 mmol) and in 10 mL of absolute ethanol was refluxed for 3 h. The formed precipitate was filtered, washed with water, dried, and recrystallized from dioxane to give buff crystals in yield 60%, m.p 279 – 280 °C. IR (KBr): 3350 (NH), 2200 (CN), 1700 (C = O), 1645 (C = N), 1620 (C = N) cm^−1^. ^1^H NMR (DMSO - d6) δ: 1.05 (s, 3H, CH_3_), 4.08 (s, 2H, CH_2_), 8.26–7.12 (m, 13H, Ar–H, thiophene, pyridine and CH aliphatic), 8.96 (s, 1H, NH), 12.58 (s, 1H, NH) ppm. ^13^C NMR (DMSO): δ = 163.09 (C=O), 152.50 (C=N), 145.93 (C), 145.54 (CH), 144.41 (C), 143.15 (CH), 138.24 (C), 130.13 (C), 129.10 (CH), 128.13, 127.88, 127.47 (3CH), 125.96 (2CH), 112.49 (CN), 101.86 (CH), 56.49 (OCH_2_), 21.24 (CH_3_) ppm. MS: m/z (%): 518 [M + , 15], 262 (100).Anal. Calc.(%) for C_25_H_19_N_5_O_4_S_2_.C,58.01; H, 3.70;N, 13.53; S, 12.39. Found C,58.07;H, 3.67; N, 13.55; S, 12.34.

### In vitro cytotoxic activity

#### Cell culture

Breast carcinoma (MCF-7) human cell line was obtained from the American type culture collection (ATCC). Cells were maintained in RPMI-1640 supplemented with (100 μg/mL); penicillin (100 units/mL) and heat-inactivated fetal bovine serum (10% v/v) in a humidified, 5% (v/v) CO_2_ atmosphere at 37° [31, 32].

#### Cytotoxicity assay

The cytotoxicity of the chemical compounds was evaluated against (MCF-7) human tumor cell using Sulphorhodamine B assay (SRB) in King Khalid University, biology department. 80% confluency growing cells were trypsinized and cultured in a 96 well tissue culture plate for 24 h before treatment with the chemical compounds. Cells were exposed to the six different concentrations of each compound (0.01, 0.1, 1, 10, and 1000 µg/ml); untreated cells (control) were added. The cells were incubated with the concentrations for 72 h and subsequently fixed with TCA (10% w/v) for 1 h at 4 °C. After several washings, cells were stained by 0.4% (w/v) SRB solution for 10 min in dark place. Excess stain was washed with 1% (v/v) glacial acetic acid. After drying overnight, the SRB-stained cells were dissolved with Tris–HCl and the color intensity was measured in microplate reader at 540 nm. The relation between viability percentage of each tumor cell line and compounds concentrations was analyzed to get the IC_50_ (dose of the drug which reduces survival to 50%) using Sigma Plot 12.0 software [33].

#### Acridine orange/ethidium bromide staining for detection of early and late apoptotic cells

DNA binding dyes Acridine orange (AO) and Ethidium bromide (EtBr), were used for the morphological detection of viable, apoptotic and necrotic cells. AO is taken up by both non-viable and viable cells that emit green fluorescence when intercalated into DNA. EtBr is taken up only by nonviable cells whereas; it is excluded by viable cells and emits red fluorescence by intercalation into DNA. Cells were seeded on cover slide inside six well plates. Cells were incubated in CO_2_ incubator with 37 °C temperature and 5% CO_2_ for 24 h then treated with IC_50_s concentration of the chemical compounds and incubated for 48 h. Cells were washed with cold PBS 1× for three times. Cells were stained with a mixture Acridine Orange 100 μg/ml/Ethidium Bromide (AO/EB) 100 μg/ml in PBS 1x with 10% FBS on each well and then incubated for 5 min in RT. The cover slides with cultured stained cells were transfer immediately to new slides and the cells were ready to be visualized by the blue filter of the fluorescence microscope [34, 35].

## Data Availability

All the data supporting findings are contained within the manuscript.

## Abbreviations

MCF-7: Breast cancer cell line
SAR: Structure–activity relationship
CAN: Ceric ammonium nitrate
1H NMR: Nuclear magnetic resonance spectroscopy
SRB: Sulforhodamine B
IC_50_: The half maximal inhibitory concentration (IC_50_)
AO/EtBr: Acridine Orange Ethidium Bromide
TLC: Thin-layer chromatography
The IR spectra: Infrared spectroscopy
KBR: Potassium bromide
TMS: Tetramethylsilane
GC/MS: Gas chromatography–mass spectrometry
^13^C NMR: Carbon-13 Nuclear Magnetic Resonance Spectroscopy
DMSO: Dimethyl Sulfoxide
ATCC: American type culture collection
SRB: Sulphorhodamine B assay
AO: Acridine orange
EtBr: Ethidium bromide
FBS: Fetal bovine serum
PBS: Phosphate-Buffered Saline
